# Cardiac magnetic resonance research advances in myocardial fibrosis of hypertrophic cardiomyopathy

**DOI:** 10.3389/fcvm.2025.1684960

**Published:** 2025-11-21

**Authors:** Yujian Liu, Minli Lv, Jianquan Zhong, Yuan Li

**Affiliations:** 1 Department of Radiology, Zigong First People’s Hospital, Zigong, Sichuan, China; 2 Department of Ultrasound, Zigong Fourth People’s Hospital, Zigong, Sichuan, China

**Keywords:** hypertrophic cardiomyopathy (HCM), myocardial fibrosis, cardiac magnetic resonance imaging (CMR), late gadolinium enhancement (LGE), T1 mapping, extracellular volume fraction (ECV)

## Abstract

Hypertrophic cardiomyopathy (HCM) is a common inherited myocardial disorder characterized by left ventricular wall thickening, cardiomyocyte disarray, and varying degrees of interstitial and replacement fibrosis. Myocardial fibrosis plays a central role in the pathological progression of HCM, directly contributing to ventricular remodeling, diastolic dysfunction, and electrical instability and serving as a key mediator of adverse clinical outcomes such as arrhythmias, heart failure, and sudden cardiac death. In recent years, cardiac magnetic resonance imaging (CMR) has been widely adopted for the non-invasive detection and quantification of myocardial fibrosis in patients with HCM due to its high spatial resolution, multiparametric assessment capabilities, and excellent tissue specificity, demonstrating significant value in structural evaluation, risk stratification, and clinical decision-making. This review systematically summarizes the key research advances in recent years regarding the assessment of myocardial fibrosis in HCM using CMR, with a particular focus on the clinical applications and research frontiers of multiparametric imaging techniques such as late gadolinium enhancement (LGE), T1 mapping, and extracellular volume fraction (ECV) in fibrosis quantification, microstructural identification, and prognostic evaluation. Additionally, it explores the current challenges in imaging standardization, parameter stability, and multicenter validation, while also envisioning future development trends involving integration with artificial intelligence, multimodal imaging, and molecular biology in patients with HCM. The aim is to provide systematic academic references for mechanistic research and personalized management of HCM fibrosis.

## 1 Introduction

HCM is an autosomal dominant inherited cardiomyopathy with an incidence of approximately 1/500, making it one of the most common primary cardiomyopathies in clinical practice (1–3). The histological features of HCM include disordered arrangement of cardiomyocytes, interstitial hyperplasia, vascular lesions, and myocardial fibrosis. The pathological types can manifest as focal replacement fibrosis and extensive interstitial diffuse fibrosis (4, 5). Among these, myocardial fibrosis not only serves as an important basis for myocardial remodeling in HCM but is also highly correlated with reduced left ventricular compliance, electrophysiological instability, and arrhythmia. It plays a critical role in disease progression, clinical stratification, and risk assessment of sudden cardiac death (SCD) (1–4, 6).

In the field of imaging evaluation, although traditional echocardiography can be used to observe myocardial hypertrophy and functional status, it has significant limitations in identifying fibrosis at the tissue level (7). Cardiac magnetic resonance (CMR) imaging, as the only currently available technology capable of non-invasive, multiparametric, multilevel tissue imaging, has become the core tool for identifying and quantifying HCM myocardial fibrosis (1, 8). CMR can detect replacement (focal) fibrosis through late gadolinium enhancement (LGE) and achieve early identification of diffuse interstitial fibrosis through native T1 mapping and ECV. Moreover, some parameters exhibit independent correlations with prognostic indicators such as SCD, heart failure hospitalization, and functional deterioration (9–11).

In recent years, with the rapid development of imaging technology, sequences such as diffusion tensor imaging (DTI) and four-dimensional flow imaging (4D Flow) have been gradually introduced into the tissue and functional assessment of HCM. Meanwhile, CMR-based radiomics analysis and artificial intelligence (AI) modeling methods have also provided new possibilities for the automated quantification and prognostic prediction of myocardial fibrosis (9, 10). However, there is still a lack of consistency in parameter measurement, technical platforms, and quantification standards among different imaging centers at present, and some emerging methods remain in the early exploratory stage, requiring large-sample, prospective studies for validation.

Based on this, relevant literature was identified through a structured search of PubMed, Web of Science, and Embase for studies published between January 2010 and June 2025, using keywords related to “hypertrophic cardiomyopathy,” “cardiac magnetic resonance,” “fibrosis,” and “radiomics.” Only English-language, peer-reviewed articles were included to ensure coverage of validated and emerging evidence. Building upon a comprehensive analysis of previous studies, this review aims to systematically summarize the progress of CMR in evaluating myocardial fibrosis in hypertrophic cardiomyopathy (HCM), with emphasis on the imaging characteristics and prognostic value of key parameters such as late gadolinium enhancement (LGE), T1 mapping, and extracellular volume (ECV). It further explores the application potential of advanced imaging techniques—including diffusion tensor imaging (DTI), four-dimensional flow (4D Flow), and artificial intelligence-assisted radiomics—in assessing myocardial function and fibrosis burden. Finally, this review discusses current challenges in standardization and clinical translation and outlines future directions for integrating multimodal imaging with intelligent algorithms to achieve precision evaluation and individualized management of HCM.

## 2 The pathological and clinical significance of myocardial fibrosis in HCM

Myocardial fibrosis plays a critical role in the structural remodeling of HCM. It may manifest as focal replacement fibrosis, resulting from cardiomyocyte necrosis or apoptosis, or as diffuse interstitial fibrosis, driven by chronic mechanical stress, ischemic injury, or fibroblast activation induced by pro-inflammatory signaling (1). These two forms of fibrosis can coexist across different HCM subtypes and stages of disease progression, collectively forming the histopathological basis of the condition. However, their underlying mechanisms and clinical manifestations differ significantly (1, 5, 12).

Replacement fibrosis typically manifests as focal lesions formed by the deposition of type I collagen replacing dead cardiomyocytes, predominantly distributed in stress-concentrated regions such as the mid-to-lower ventricular septum, the base of the papillary muscles, and the right ventricular insertion sites. This type of fibrosis can be identified via LGE imaging, appearing as localized hyperintense signals (10, 13). In contrast, interstitial fibrosis is characterized by diffuse collagen deposition in the myocardial interstitium, often remaining “invisible” on conventional imaging in its early stages and requiring indirect quantification through parameters such as T1 mapping and ECV (14, 15). Histological studies confirm that in patients with HCM, the ratio of type I to type III collagen increases in the myocardium, while an imbalance between matrix metalloproteinases (MMPs) and their tissue inhibitors (TIMPs) leads to excessive extracellular matrix (ECM) accumulation, reduced myocardial elasticity, and disrupted mechano-electrical coupling (6, 16).

At the electrophysiological level, fibrotic regions serve as the basis for functional block and conduction abnormalities, leading to localized depolarization delay, reentrant circuit formation, and focal autonomic dysfunction in patients with HCM, thereby significantly increasing the risk of arrhythmia Both autopsy and imaging studies indicate that myocardial fibrosis is widely distributed in the myocardium of HCM patients who experience SCD, and it is particularly closely associated with non-sustained ventricular tachycardia (NSVT). Extensive research suggests that when the LGE burden reaches ≥5%, the risk of SCD rises significantly, and this has been incorporated into some risk stratification models as a key indicator to guide decision-making for implantable cardioverter-defibrillator (ICD) placement.

Moreover, the extent of myocardial fibrosis is closely associated with the phenotypic expression of HCM. Individuals with higher fibrotic burden are more prone to decreased exercise tolerance, diastolic dysfunction, heart failure-related hospitalization, and worsening functional class. In contrast, those with lower ECV and T1 values have demonstrated relatively stable clinical progression trends in multiple studies, suggesting that myocardial fibrosis may serve as a crucial stratification and risk prediction marker in clinical practice (17, 18). However, this correlation still requires comprehensive evaluation in conjunction with genotype, electrophysiological phenotype, and clinical scoring.

In summary, myocardial fibrosis not only plays a central role in the myocardial structure of HCM and the remodeling of the myocardium but also serves as a crucial intermediary mechanism determining functional impairment, electrical instability, and worsening prognosis. It may develop as early as the initial stages of the disease and is highly correlated with terminal events such as sudden death and heart failure. Non-invasive quantitative assessment based on CMR provides a new pathway for understanding its pathological progression and clinical impact, while also laying an important foundation for optimizing individualized classification and intervention strategies.

## 3 The evolution of CMR imaging techniques for myocardial fibrosis

The diagnostic techniques of CMR for myocardial fibrosis have continuously evolved over time, progressing from early-stage simple morphological observations to the current capability of precise quantitative analysis of myocardial tissue characteristics.

In the early stages, CMR primarily relied on morphological imaging to indirectly infer the possibility of myocardial fibrosis, such as observing changes in heart size, shape, and ventricular wall thickness. With technological advancements, LGE has become a crucial method for detecting myocardial fibrosis. LGE is based on the differences in the distribution and clearance rates of gadolinium contrast agents between normal and fibrotic myocardium, causing fibrotic regions to exhibit high signal intensity during the delayed phase, thereby visually displaying the location and extent of myocardial fibrosis (3, 19). For example, in patients with HCM, LGE can clearly reveal myocardial fibrosis foci, aiding in the assessment of disease severity and prognosis.

In recent years, the emergence of T1 mapping technology has brought new breakthroughs in the diagnosis of myocardial fibrosis. This technique can quantitatively measure the longitudinal relaxation time (T1 value) of the myocardium. By comparing the T1 value differences between normal and diseased myocardium, it is possible to assess changes in myocardial tissue composition, particularly demonstrating significant value in detecting diffuse myocardial fibrosis. When combined with pre- and post-contrast agent T1 value measurements, the ECV can also be calculated. ECV is considered a reliable indicator reflecting the degree of myocardial fibrosis and shows a strong correlation with histological fibrosis levels. Research indicates that in various cardiac diseases, such as HCM and dilated cardiomyopathy, T1 mapping and ECV measurements can detect early myocardial fibrosis changes, providing a basis for the early diagnosis and intervention of these conditions (14, 15).

In addition, diffusion-weighted imaging (DWI) and DTI have gradually been applied to the study of myocardial fibrosis. DWI reflects changes in the myocardial tissue structure of myocardial tissue microstructure by detecting the diffusion movement of water molecules, while DTI can further analyze the direction of water molecule diffusion on this basis, thereby evaluating the arrangement of cardiac muscle fibers and the microstructure of These techniques are expected to play an important role in the early identification of microstructural changes and disorganized fibers in within the myocardium, providing new perspectives for the early diagnosis of myocardial fibrosis.

## 4 Application of LGE technology in HCM fibrosis assessment

### 4.1 Imaging mechanism and anatomical manifestations

LGE utilizes the prolonged retention of gadolinium-based contrast agents (typically dosed at 0.1–0.2 mmol/kg) in fibrotic tissues, employing inversion recovery sequences to achieve high-contrast differentiation between pathological and normal myocardium. Sequence selection often involves TI scout localization to determine the optimal inversion time (null point) for maximizing contrast difference. Imaging is predominantly acquired in short-axis, four-chamber, and three-chamber views, with spatial resolution ranging from 1.5 to 2 mm. Anatomically, in HCM patients, LGE is frequently localized to the mid-to-lower ventricular septum, papillary muscle insertion zones, right ventricular insertion points, and regions of mechanical stress, appearing as replacement fibrosis as hyperintense signals (19, 20).

### 4.2 LGE quantification methods and load assessment

LGE quantification typically employs three main approaches: (1) visual scoring, which is simple but highly subjective, and (2) signal intensity thresholding, usually defined as the mean signal of normal myocardium plus 6 SD, which improves accuracy but depends on ROI selection; and (3) percentage measurement, calculating the LGE volume as a percentage of LV myocardium, providing a reproducible continuous index (21–23).

In recent years, deep learning and automated image processing techniques have been applied to fully automated segmentation and fibrosis detection in LGE images. Models such as ScarNet achieve Dice coefficients exceeding 0.9, significantly improving quantification efficiency and consistency (24).

Multiple meta-analyses confirm that a greater LGE burden strongly predicts sudden cardiac death and heart failure events in hypertrophic cardiomyopathy. In a cohort of 5,550 patients, every 10% increase in LGE extent was associated with a 1.56-fold higher SCD risk, and thresholds of approximately 5%–10% helped reclassify intermediate-risk patients. These findings support LGE as a validated prognostic marker now incorporated into European Society of Cardiology (ESC) and American Heart Association (AHA) guidelines for clinical decision support (25–27).

Nevertheless, despite its established role, LGE exhibits substantial inter-center variability due to differences in scanners, magnetic field strength, and contrast protocols. It also lacks sensitivity for small or diffuse fibrosis, and no universal cutoff values have been defined. Furthermore, most available studies are single-center and retrospective, which may limit reproducibility and external generalizability. Therefore, LGE findings should be interpreted in conjunction with T1 mapping or ECV to achieve a more comprehensive and reproducible fibrosis assessment. Future multicenter, prospective studies with standardized imaging protocols are warranted to establish uniform thresholds and confirm the prognostic robustness of LGE across diverse populations (28–34).

## 5 The value of T1 mapping and ECV in diffuse fibrosis assessment

### 5.1 Principles and technical overview

T1 mapping provides voxel-level quantification of myocardial tissue using sequences such as MOLLI, ShMOLLI, and SASHA. MOLLI [e.g., 5(3)3] balances accuracy and scan time, ShMOLLI enables shorter breath-holds, and SASHA (saturation recovery) offers more accurate T1 estimation but is more sensitive to noise. Multicenter studies have shown sequence- and vendor-related differences in native T1 and ECV measurements; however, repeat-scan stability—particularly for ECV—is acceptable, with variability typically between 0.01 and 0.02 (35–37).

ECV is calculated from pre- and post-contrast T1 values of blood and myocardium with hematocrit using the formula ECV = (1 − Hct) × (ΔR1_myocardium/ΔR1_blood). This parameter represents the volumetric fraction of ECM expansion and provides a quantitative index of myocardial fibrotic burden (38–40).

### 5.2 Identification, prognostic implications, and clinical utility

In hypertrophic cardiomyopathy, T1 mapping and ECV enable identification of diffuse interstitial fibrosis beyond the focal replacement fibrosis detected by LGE and provide incremental prognostic information, especially in LGE-negative patients. Li et al. (20) reported that increased ECV independently predicted heart failure hospitalization and mortality (HR ≈ 1.3 per 3% increase, *P* < 0.001), even after adjusting for LGE. Native T1 has also been identified as an independent predictor of major adverse cardiovascular events, particularly in low-risk cohorts where guideline risk scores have limited discrimination (41).

Moreover, T1/ECV phenotypes differ between obstructive and non-obstructive HCM, and higher fibrosis burden correlates with symptom severity, reduced exercise capacity, and elevated NT-proBNP levels (42). Stress- and rest-state T1 mapping can further reflect microvascular perfusion deficits, reinforcing their role as complementary rather than replacement markers for LGE (43).

### 5.3 Limitations, standardization needs, and future work

Despite their advantages, T1 and ECV mapping techniques face persistent challenges in reproducibility and cross-center comparability. Variations in sequence design (MOLLI, ShMOLLI, SASHA), magnetic field strength (1.5 T vs. 3 T), and vendor-specific calibration, as well as patient-related factors such as motion artifacts, heart rate dependency, and B1 inhomogeneity, contribute to measurement inconsistency. Although ECV calculation can partially compensate for these effects, inter-sequence and inter-vendor bias still restricts direct comparability, and no universally accepted diagnostic or prognostic thresholds have been established (44–46).

Furthermore, most current studies remain small, retrospective, and single-center, resulting in heterogeneity of reported values and limited external generalizability. These methodological differences must be considered when interpreting the clinical utility of T1 and ECV parameters.

Future work should focus on improving acquisition stability, motion correction, and signal standardization within sites, while fostering reproducible analytic pipelines. Large-scale, prospectively acquired datasets will be essential to refine measurement precision and enable consistent longitudinal and inter-scanner comparisons (46, 47).

## 6 Exploration of emerging imaging technologies in HCM fibrosis research

### 6.1 DTI for myocardial microstructure

DTI quantifies myocardial microstructure through diffusion parameters such as fractional anisotropy (FA), mean diffusivity (MD), and the second eigenvector angle (E2A). *In vivo* comparisons by Nguyen et al. (48) showed increased MD and E2A with reduced FA in HCM compared with healthy controls (MD 1.52 vs. 1.47 × 10^−3^ mm^2^/s; FA 0.30 vs. 0.36; *P* < 0.05), reflecting disorganized sheetlet architecture. Elevated E2A in hypertrophic segments (66.8 vs. 51.2) correlated with diastolic dysfunction and microstructural remodeling (49). DTI abnormalities also align with electrophysiologic instability: persistence of E2A during diastole indicates impaired relaxation and potential reentrant substrate formation. Although sample sizes remain small, DTI offers a novel window into microscopic fibrosis and could complement conventional imaging once standardized scanning and fusion protocols are established (50).

### 6.2 Four-dimensional flow imaging (4D flow) and hemodynamics in HCM

4D flow CMR quantifies multidirectional intracardiac hemodynamics, allowing assessment of left ventricular outflow tract (LVOT) obstruction and vortex dynamics. van Ooij et al. (51) demonstrated higher LVOT peak gradients (21 ± 16 mmHg vs. 9 ± 2 mmHg) and energy dissipation rates (3.8 ± 2.5 mW vs. 1.5 ± 0.7 mW, *P* < 0.005) in HCM, correlating with ECV (*r*^2^ ≈ 0.45). These alterations suggest that abnormal shear stress and vortex morphology contribute to fibrosis progression. Despite technical challenges—long scan times and heavy post-processing—deep learning tools (e.g., 4DFlowNet) are reducing scan time and improving quantification, supporting integration into refined functional–fibrotic evaluation frameworks (52).

### 6.3 Prospects of combined evaluation with PET/MR and T2 mapping

PET/MR combines anatomical and metabolic imaging for early fibrosis detection. In a 2023 radiology study, ^18^F-FAPI PET/MR revealed increased myocardial uptake correlating with the ESC 5-year SCD risk score (*r* = 0.32, *P* < 0.001) in 50 HCM patients, suggesting fibroblast activation precedes LGE-visible fibrosis (53). ^18^F-FDG PET/MR and perfusion tracers (O-15 water, ^13^N-ammonia) confirm frequent microvascular dysfunction overlapping with fibrotic regions (54–56).

T2 mapping quantifies myocardial water content and detects inflammatory or edematous components accompanying fibrosis. Goldie et al. (57) observed significantly elevated T2 in LGE-positive zones (*P* < 0.01), identifying active remodeling. However, variability across field strengths and protocols limits reproducibility.

### 6.4 Summary and outlook

Emerging techniques such as DTI, 4D Flow, PET/MR, and T2 mapping enrich understanding of HCM fibrosis from structural, hemodynamic, metabolic, and inflammatory perspectives. While early evidence demonstrates diagnostic and prognostic potential, clinical validation remains limited. Future priorities include standardized acquisition, multicenter trials, and integration with established CMR parameters (LGE, T1/ECV) to develop clinically applicable, multimodal fibrosis assessment pathways.

## 7 Radiomics and artificial intelligence in automated fibrosis analysis

### 7.1 Radiomics for quantitative fibrosis assessment

Radiomics enables the extraction of high-dimensional features from CMR sequences such as LGE, T1 mapping, and cine imaging to quantify myocardial heterogeneity. Studies have demonstrated its ability to distinguish fibrotic from non-fibrotic myocardium and to support objective fibrosis quantification. In a *Journal of Cardiovascular Magnetic Resonance* (JCMR) study, radiomic models derived from cine and LGE sequences achieved an AUC of 0.89 for fibrosis detection, while another study of 273 HCM patients confirmed excellent feature reproducibility [intraclass correlation coefficient (ICC) > 0.85] and effective differentiation between LGE-positive and LGE-negative phenotypes (58, 59). Integrating radiomic features with quantitative parameters such as ECV or native T1 further improves risk prediction, increasing model discrimination and reclassification performance in both HCM and related cardiomyopathies (60).

### 7.2 Deep learning and AI-assisted modeling

Convolutional neural networks (CNNs) and anatomically informed deep learning models enable automated myocardial and fibrosis segmentation with near-expert accuracy. Multisequence networks such as MyoPS-Net have achieved high Dice coefficients and <2% difference in scar burden compared with expert manual labeling (61, 62). Multimodal AI models combining radiomics, ECV, and clinical variables have shown improved prediction of arrhythmic events and sudden cardiac death, enhancing the C-statistic of established ESC and AHA risk models (59, 60).

### 7.3 Challenges and clinical translation

Despite rapid progress, most radiomics and AI studies remain single-center and retrospective, limiting generalizability. Feature stability, biological interpretability, and the “black-box” nature of AI models restrict clinical acceptance. Standardized acquisition, reproducible feature extraction, and regulatory validation are essential before clinical adoption (58, 60, 61). Future work should prioritize multicenter prospective studies and development of interpretable, guideline-compatible AI tools that complement existing fibrosis markers (LGE, T1, ECV) to facilitate precise and automated HCM fibrosis assessment (63–65).

## 8 Current challenges and future directions

**Figure 1:**
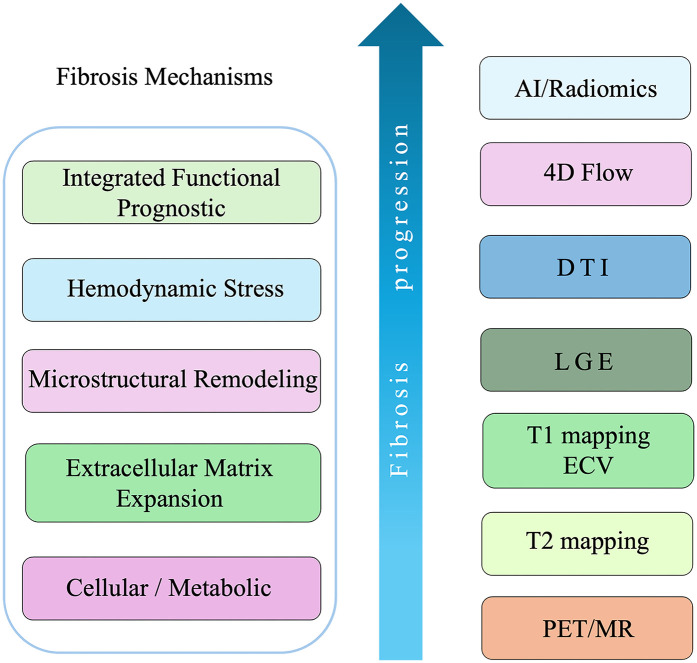
Schematic overview of fibrosis mechanisms and corresponding CMR modalities in HCM.

A schematic overview summarizing the multilevel mechanisms of myocardial fibrosis and corresponding CMR modalities is presented in Figure 1, integrating the structural, functional, and molecular imaging perspectives discussed in Sections 4–7 (Figure 1).

The diagram illustrates the progressive mechanisms of myocardial fibrosis and their corresponding CMR assessment techniques.

On the left, fibrosis evolves through five biological levels:

1. Cellular/metabolic activation—initial myocyte stress, inflammation, and fibroblast activation
2. ECM expansion—accumulation of collagen and interstitial remodeling
3. Microstructural remodeling—disorganization of myofibril alignment and increased anisotropy
4. Hemodynamic stress—altered ventricular flow, pressure overload, and energetic inefficiency
5. Integrated functional/prognostic remodeling—global systolic–diastolic impairment and arrhythmogenic substrate formation

On the right, each CMR modality corresponds to these stages: PET/MR and T2 mapping detect early cellular and inflammatory changes; T1 mapping and ECV quantify ECM expansion; LGE and DTI characterize replacement fibrosis and microstructural disarray; 4D Flow visualizes hemodynamic abnormalities; and AI/radiomics integrate multimodal features for functional and prognostic prediction.

The upward arrow indicates the continuum of fibrosis progression from early metabolic activity to advanced functional remodeling.

### 8.1 Standardization and cross-center quality control

As discussed in Section 5.3, technical and physiological factors contribute to substantial variability in T1 and ECV measurements. Beyond single-scanner optimization, the key challenge now lies in achieving reproducibility across centers and vendors. Differences in pulse-sequence design, field strength, and post-processing software continue to hinder inter-site comparison, limiting the use of absolute T1 and ECV values for risk stratification and multicenter research (63, 64).

To address these issues, international initiatives such as the CMR-T1MES phantom program and vendor-neutral calibration frameworks are developing reference phantoms and harmonized quality control (QC) protocols that allow cross-platform comparison of mapping values. These efforts emphasize periodic phantom scanning, centralized data repositories, and automated QC pipelines to minimize scanner-specific bias and ensure traceability of measurement standards (63–65).

Moving forward, the field should establish multicenter reference databases with harmonized acquisition and analysis protocols, define *z*-score-based normalization relative to field strength and vendor, and adopt shared open-source QC software for routine implementation. These strategies will enable evidence-based thresholds, improve data comparability, and accelerate clinical translation of quantitative fibrosis imaging in hypertrophic cardiomyopathy.

### 8.2 From multimodal research to translational integration

Most current studies linking CMR fibrosis markers to adverse outcomes such as sudden cardiac death, heart failure, or ICD therapy remain single-center and retrospective. Prospective, multicenter studies with outcome validation are required to confirm prognostic utility and define clinical indications. Future integration should combine CMR metrics with genomics, circulating biomarkers, and AI-based models to enhance individualized risk prediction. Recent nature and radiology reports have demonstrated that fusing LGE/ECV parameters with genomic or transcriptomic profiles via machine-learning algorithms markedly improves fibrosis and outcome prediction, highlighting the promise of multimodal, data-driven precision imaging (53, 57, 66–69).

### 8.3 Clinical translation and implementation pathways

Although LGE, T1, and ECV are independently associated with outcomes in HCM, they are not yet formally embedded in guideline-based decision tools. Current ESC and AHA/American College of Cardiology (ACC) risk models rely mainly on clinical variables, and CMR findings are recommended as adjuncts within shared decision-making rather than stand-alone triggers for ICD implantation (57, 70). Meta-analyses indicate that an LGE threshold of approximately 10% of LV mass may reclassify intermediate-risk patients, yet cutoffs vary and require external validation (25, 42, 71). Similarly, native T1 and ECV offer incremental prognostic information, especially in LGE-negative patients, but remain investigational pending multicenter evidence.

CMR also provides non-invasive monitoring of therapeutic response, exemplified by imaging-documented reverse remodeling during cardiac myosin-inhibitor therapy (e.g., mavacamten). However, correlations between imaging regression and hard outcomes remain uncertain, so fibrosis burden should be considered an exploratory surrogate endpoint (72–74).

### 8.4 Outlook

Moving forward, standardized acquisition protocols, harmonized post-processing, and prospective multicenter trials are prerequisites for clinical implementation. Integrating CMR-derived fibrosis markers with genetic, molecular, and clinical variables will enable refined, individualized management of HCM. Ultimately, establishing guideline-compatible, outcome-validated imaging frameworks will transform fibrosis assessment from descriptive imaging into actionable precision medicine (75).

### 8.5 Comparative summary of multiparametric CMR biomarkers

The following table summarizes mechanisms, advantages, limitations, and clinical applications of key CMR biomarkers used in HCM (Table 1).

**Table 1:** Concise comparative summary of multiparametric CMR biomarkers in HCM.

CMR modality	Mechanism/target	Strengths	Limitations	Clinical role in HCM
LGE	Gadolinium distribution and delayed washout in scar (replacement fibrosis)	Robust for replacement fibrosis Strong prognostic association (SCD/HF) Widely available	Low sensitivity for diffuse fibrosis Inter-center variability Cutoff heterogeneity Partial-volume/slice coverage	Adjunctive risk stratification ICD decision support (not stand-alone)
T1 mapping/ECV	Native T1 and ECV quantify interstitial matrix expansion (diffuse fibrosis)	Detects diffuse fibrosis Incremental prognostic value beyond LGE Useful when LGE is low/absent	Sequence/vendor dependence (MOLLI/shmolli/SASHA) Field strength reference ranges Motion/B1 effects No universal cutoffs	Complements LGE for risk HF/arrhythmia prediction Therapy monitoring (exploratory)
T2 mapping	Myocardial water content (edema/inflammation)	Non-contrast Flags active inflammatory/edematous changes	Susceptible to B0/B1 and motion Limited multicenter outcomes data	Stage activity assessment Adjunct to fibrosis imaging
DTI	Fiber/sheetlet architecture (helix angle, E2A, FA) → microstructural disarray	Direct microstructural insight; Mechanistic link to arrhythmogenesis	Technically demanding Gating/motion correction required Small-cohort evidence	Early remodeling assessment Risk refinement (emerging)
4D Flow	Time-resolved 3D velocity field (LVOT jet, vortices, KE/EL, WSS)	Quantifies hemodynamics and LVOT obstruction Links flow to tissue metrics (T1/ECV)	Long scan and post-processing Advanced software; need standardization/QA	Phenotyping obstructive HCM Pathophysiology Septal-reduction planning (emerging)
PET/MR	Molecular tracers (e.g., ^18^F-FDG, ^68^Ga/^18^F-FAPI) + MR anatomy	Detects metabolic/fibroblast activity pre-scar Multiparametric	Cost/availability/radiation Registration complexity Limited outcomes-linked data	Early molecular assessment Therapy timing/monitoring (exploratory)

Report LGE as %LV and specify quantification (e.g., 6-SD, FWHM). ECV requires contemporaneous hematocrit; use site/field strength-specific reference ranges or z-scores.

ECV, extracellular volume fraction; DTI, diffusion tensor imaging; LVOT, left ventricular outflow tract; ICD, implantable cardioverter-defibrillator; SCD, sudden cardiac death; HF, heart failure; KE, kinetic energy; EL, energy loss; WSS, wall shear stress; FA, fractional anisotropy.

## 9 Conclusion

CMR has become the reference standard for non-invasive evaluation of myocardial fibrosis in hypertrophic cardiomyopathy. LGE provides a well-validated marker of replacement fibrosis with established prognostic value for sudden cardiac death and heart failure, whereas T1 mapping and ECV extend assessment to diffuse fibrosis and improve risk stratification.

However, parameter variability across sequences and scanners limits reproducibility, and current data remain largely retrospective. AI-based radiomics, PET/MR, DTI, and 4D flow offer new insights into tissue microstructure and metabolism but remain investigational (28, 76, 77).

Future work should prioritize standardization of imaging protocols, multicenter prospective validation, and multimodal integration with genetics and biomarkers to define evidence-based thresholds and guide individualized management of HCM fibrosis.

## References

[1] Semsarian C Ingles J Maron MS Maron BJ. New perspectives on the prevalence of hypertrophic cardiomyopathy. J Am Coll Cardiol. (2015) 65(12):1249–54. 10.1016/j.jacc.2015.01.019 25814232

[2] Dias KA Link MS Levine BD. Exercise training for patients with hypertrophic cardiomyopathy: JACC review topic of the week. J Am Coll Cardiol. (2018) 72(10):1157–65. 10.1016/j.jacc.2018.06.054 30165987

[3] Maron BJ Maron MS. Hypertrophic cardiomyopathy. Lancet. (2013) 381(9862):242–55. 10.1016/S0140-6736(12)60397-3 22874472

[4] Batzner A Seggewiss H. Hypertrophic cardiomyopathy. Herz. (2020) 45(3):233–42. 10.1007/s00059-020-04899-y 32185419

[5] BaniHani HA Khaled LH Al Sharaa NM Al Saleh RA Bin Ghalaita AK Bin Sulaiman AS Causes, diagnosis, treatment, and prognosis of cardiac fibrosis: a systematic review. Cureus. (2025) 17(3):e81264. 10.7759/cureus.81264 40291288 PMC12032538

[6] Bi X Yang C Song Y Yuan J Cui J Hu F Matrix metalloproteinases increase because of hypoperfusion in obstructive hypertrophic cardiomyopathy. Ann Thorac Surg. (2021) 111(3):915–22. 10.1016/j.athoracsur.2020.05.156 32738221

[7] Park J Yoon YE Jang Y Jung T Jeon J Lee SA Novel deep learning framework for simultaneous assessment of left ventricular mass and longitudinal strain: clinical feasibility and validation in patients with hypertrophic cardiomyopathy. J Echocardiogr. (2025). 10.1007/s12574-025-00694-y 40650815

[8] Bluemke DA Lima JAC. Using MRI to probe the heart in hypertrophic cardiomyopathy. Radiology. (2020) 294(2):287–8. 10.1148/radiol.2019192370 31770077

[9] Hanneman K. The clinical significance of cardiac MRI late gadolinium enhancement in hypertrophic cardiomyopathy. Radiology. (2022) 302(2):307–8. 10.1148/radiol.2021212214 34726540

[10] Liu J Zhao S Yu S Wu G Wang D Liu L Patterns of replacement fibrosis in hypertrophic cardiomyopathy. Radiology. (2022) 302(2):298–306. 10.1148/radiol.2021210914 34726536

[11] Kamp NJ Chery G Kosinski AS Desai MY Wazni O Schmidler GS Risk stratification using late gadolinium enhancement on cardiac magnetic resonance imaging in patients with hypertrophic cardiomyopathy: a systematic review and meta-analysis. Prog Cardiovasc Dis. (2021) 66:10–6. 10.1016/j.pcad.2020.11.001 33171204

[12] Frangogiannis NG. Cardiac fibrosis. Cardiovasc Res. (2021) 117(6):1450–88. 10.1093/cvr/cvaa324 33135058 PMC8152700

[13] Dalsania AK Park CM Nagraj S Lorenzatti D Filtz A Weissler-Snir A A practical approach to multimodality imaging in hypertrophic cardiomyopathy. J Clin Med. (2025) 14(8):2606. 10.3390/jcm14082606 40283436 PMC12027606

[14] Nguyen C Lu M Fan Z Bi X Kellman P Zhao S Contrast-free detection of myocardial fibrosis in hypertrophic cardiomyopathy patients with diffusion-weighted cardiovascular magnetic resonance. J Cardiovasc Magn Reson. (2015) 17:107. 10.1186/s12968-015-0214-1 26631061 PMC4668676

[15] Wang K Zhang W Li S Jin H Jin Y Wang L Noncontrast T1rho dispersion imaging is sensitive to diffuse fibrosis: a cardiovascular magnetic resonance study at 3T in hypertrophic cardiomyopathy. Magn Reson Imaging. (2022) 91:1–8. 10.1016/j.mri.2022.05.001 35525524

[16] Serraino GF Jiritano F Costa D Ielapi N Napolitano D Mastroroberto P Metalloproteinases and hypertrophic cardiomyopathy: a systematic review. Biomolecules. (2023) 13(4):665. 10.3390/biom13040665 37189412 PMC10136246

[17] Karabinowska-Malocha A Dziewiecka E Banys P Urbanczyk-Zawadzka M Krupinski M Mielnik M The relationship between cardiac magnetic resonance-assessed replacement and interstitial fibrosis and ventricular arrhythmias in hypertrophic cardiomyopathy. J Pers Med. (2022) 12(2):294. 10.3390/jpm12020294 35207782 PMC8876292

[18] Dong Y Yang W Chen C Ji J Zheng W Zhang F Validation of the 2020 AHA/ACC risk stratification for sudden cardiac death in Chinese patients with hypertrophic cardiomyopathy. Front Cardiovasc Med. (2021) 8:691653. 10.3389/fcvm.2021.691653 34485400 PMC8415905

[19] Pan JA. Late gadolinium enhancement in hypertrophic cardiomyopathy: is there more to it than size? JACC Cardiovasc Imaging. (2024) 17(5):498–500. 10.1016/j.jcmg.2023.11.013 38180415 PMC11227108

[20] Li Y Liu X Yang F Wang J Xu Y Fang T Prognostic value of myocardial extracellular volume fraction evaluation based on cardiac magnetic resonance T1 mapping with T1 long and short in hypertrophic cardiomyopathy. Eur Radiol. (2021) 31(7):4557–67. 10.1007/s00330-020-07650-7 33449190

[21] Liu D Ma X Liu J Zhao L Chen H Xu L Quantitative analysis of late gadolinium enhancement in hypertrophic cardiomyopathy: comparison of diagnostic performance in myocardial fibrosis between gadobutrol and gadopentetate dimeglumine. Int J Cardiovasc Imaging. (2017) 33(8):1191–200. 10.1007/s10554-017-1101-7 28289991

[22] van der Velde N Hassing HC Bakker BJ Wielopolski PA Lebel RM Janich MA Improvement of late gadolinium enhancement image quality using a deep learning-based reconstruction algorithm and its influence on myocardial scar quantification. Eur Radiol. (2021) 31(6):3846–55. 10.1007/s00330-020-07461-w 33219845 PMC8128730

[23] Jada L Holtackers RJ Martens B Nies H Van De Heyning CM Botnar RM Quantification of myocardial scar of different etiology using dark- and bright-blood late gadolinium enhancement cardiovascular magnetic resonance. Sci Rep. (2024) 14(1):5395. 10.1038/s41598-024-52058-8 38443457 PMC10914833

[24] Navidi Z Sun J Chan RH Hanneman K Al-Arnawoot A Munim A Interpretable machine learning for automated left ventricular scar quantification in hypertrophic cardiomyopathy patients. PLOS Digit Health. (2023) 2(1):e0000159. 10.1371/journal.pdig.0000159 36812626 PMC9931226

[25] Kiaos A Daskalopoulos GN Kamperidis V Ziakas A Efthimiadis G Karamitsos TD. Quantitative late gadolinium enhancement cardiac magnetic resonance and sudden death in hypertrophic cardiomyopathy: a meta-analysis. JACC Cardiovasc Imaging. (2024) 17(5):489–97. 10.1016/j.jcmg.2023.07.005 37632503

[26] Prasad SK Akbari T Bishop MJ Halliday BP Leyva-Leon F Marchlinski F. Late gadolinium enhancement imaging and sudden cardiac death. Eur Heart J. (2025) 46(36):3555–68. 10.1093/eurheartj/ehaf464 40664474 PMC12450523

[27] Weng Z Yao J Chan RH He J Yang X Zhou Y Prognostic value of LGE-CMR in HCM: a meta-analysis. JACC Cardiovasc Imaging. (2016) 9(12):1392–402. 10.1016/j.jcmg.2016.02.031 27450876

[28] Karur GR Aneja A Stojanovska J Hanneman K Latchamsetty R Kersting D Imaging of cardiac fibrosis: an update, from the AJR special series on imaging of fibrosis. AJR Am J Roentgenol. (2024) 222(6):e2329870. 10.2214/AJR.23.29870 37753860

[29] Gupta S Ge Y Singh A Grani C Kwong RY. Multimodality imaging assessment of myocardial fibrosis. JACC Cardiovasc Imaging. (2021) 14(12):2457–69. 10.1016/j.jcmg.2021.01.027 34023250

[30] Hoh T Margolis I Weine J Joyce T Manka R Weisskopf M Impact of late gadolinium enhancement image acquisition resolution on neural network based automatic scar segmentation. J Cardiovasc Magn Reson. (2024) 26(1):101031. 10.1016/j.jocmr.2024.101031 38431078 PMC10981112

[31] Cui Q Yu J Ge X Gao G Liu Y He Q Diagnostic value of LGE and T1 mapping in multiple myeloma patients’ heart. BMC Cardiovasc Disord. (2024) 24(1):230. 10.1186/s12872-024-03895-y 38678215 PMC11055279

[32] Oliveira Antunes M Fernandes F Arteaga-Fernandez E Alvarez Ramires FJ Machado Correia V Novaes Cardoso J Validation of ACC/AHA and ESC sudden cardiac death risk guidelines in diverse hypertrophic cardiomyopathy cohort: stratification HCM study. Glob Heart. (2024) 19(1):94. 10.5334/gh.1380 39713197 PMC11661054

[33] Ravi SN O’Shea M Baqal O Fatunde OA Savic J Green DB The incremental role of late gadolinium enhancement in risk stratifying high risk patients with hypertrophic cardiomyopathy. Am Heart J. (2025) 289:28–37. 10.1016/j.ahj.2025.04.030 40324572

[34] Asfour I Karim S Tabraiz SA Chahal A Khanji MY Sherif AA Late gadolinium enhancement and electrocardiographic associations in hypertrophic cardiomyopathy. Ann Noninvasive Electrocardiol. (2025) 30(4):e70077. 10.1111/anec.70077 40464065 PMC12134768

[35] Roujol S Weingartner S Foppa M Chow K Kawaji K Ngo LH Accuracy, precision, and reproducibility of four T1 mapping sequences: a head-to-head comparison of MOLLI, ShMOLLI, SASHA, and SAPPHIRE. Radiology. (2014) 272(3):683–9. 10.1148/radiol.14140296 24702727 PMC4263641

[36] Heidenreich JF Weng AM Donhauser J Greiser A Chow K Nordbeck P T1- and ECV-mapping in clinical routine at 3T: differences between MOLLI, ShMOLLI and SASHA. BMC Med Imaging. (2019) 19(1):59. 10.1186/s12880-019-0362-0 31370821 PMC6676542

[37] Child N Suna G Dabir D Yap ML Rogers T Kathirgamanathan M Comparison of MOLLI, shMOLLLI, and SASHA in discrimination between health and disease and relationship with histologically derived collagen volume fraction. Eur Heart J Cardiovasc Imaging. (2018) 19(7):768–76. 10.1093/ehjci/jex309 29237044

[38] Martuszewski A Paluszkiewicz P Poreba R Gac P. Clinical significance of extracellular volume of myocardium (ECV) assessed by computed tomography: a systematic review and meta-analysis. J Clin Med. (2025) 14(6):2066. 10.3390/jcm14062066 40142874 PMC11942809

[39] Kim PK Hong YJ Sakuma H Chawla A Park JK Park CH Myocardial extracellular volume fraction and change in hematocrit level: mR evaluation by using T1 mapping in an experimental model of Anemia. Radiology. (2018) 288(1):93–8. 10.1148/radiol.2018171342 29613847

[40] Cundari G Galea N Mergen V Alkadhi H Eberhard M. Myocardial extracellular volume quantification with computed tomography-current status and future outlook. Insights Imaging. (2023) 14(1):156. 10.1186/s13244-023-01506-6 37749293 PMC10519917

[41] Qin L Min J Chen C Zhu L Gu S Zhou M Incremental values of T1 mapping in the prediction of sudden cardiac death risk in hypertrophic cardiomyopathy: a comparison with two guidelines. Front Cardiovasc Med. (2021) 8:661673. 10.3389/fcvm.2021.661673 34169099 PMC8217449

[42] Zdebik N Poreba R Gac P. Importance of T1-mapping sequence in patients with hypertrophic cardiomyopathy without foci of non-ischemic myocardial injury in late gadolinium enhancement sequence. Biomedicines. (2024) 12(6):1330. 10.3390/biomedicines12061330 38927537 PMC11202304

[43] Kosuge H Hachiya S Fujita Y Hida S Chikamori T. Potential of non-contrast stress T1 mapping for the assessment of myocardial injury in hypertrophic cardiomyopathy. J Cardiovasc Magn Reson. (2023) 25(1):53. 10.1186/s12968-023-00966-5 37759307 PMC10536753

[44] Hanania E Zehavi-Lenz A Volovik I Link-Sourani D Cohen I Freiman M. MBSS-T1: model-based subject-specific self-supervised motion correction for robust cardiac T1 mapping. Med Image Anal. (2025) 102:103495. 10.1016/j.media.2025.103495 39987819

[45] Lee W Han PK Marin T Mounime IBG Vafay Eslahi S Djebra Y Free-breathing 3D cardiac extracellular volume (ECV) mapping using a linear tangent space alignment (LTSA) model. Magn Reson Med. (2025) 93(2):536–49. 10.1002/mrm.30284 39402014 PMC11606777

[46] Zhong Y Li C Yu Y Du Y Bai Y Wang X Evaluation the relationship between myocardial fibrosis and left ventricular torsion measured by cardiac magnetic resonance feature-tracking in hypertrophic cardiomyopathy patients with preserved ejection fraction. Int J Cardiovasc Imaging. (2024) 40(4):921–30. 10.1007/s10554-024-03061-7 38448705

[47] Zhi Y Zhang TY Gui FD Wen M Gao LC Long YT Myocardial fibrosis evaluated by T1 mapping and its relationship to left ventricular hypertrophy, strain, and T2 value in hypertrophic cardiomyopathy without late gadolinium enhancement. J Thorac Imaging. (2025). 10.1097/RTI.0000000000000862 41084187

[48] Das A Kelly C Teh I Nguyen C Brown LAE Chowdhary A Phenotyping hypertrophic cardiomyopathy using cardiac diffusion magnetic resonance imaging: the relationship between microvascular dysfunction and microstructural changes. Eur Heart J Cardiovasc Imaging. (2022) 23(3):352–62. 10.1093/ehjci/jeab210 34694365 PMC8863073

[49] Khalique Z Ferreira PF Scott AD Nielles-Vallespin S Martinez-Naharro A Fontana M Diffusion tensor cardiovascular magnetic resonance in cardiac amyloidosis. Circ Cardiovasc Imaging. (2020) 13(5):e009901. 10.1161/CIRCIMAGING.119.009901 32408830 PMC7255887

[50] Dong Z Tang Y Sun P Yin G Zhao K Ma X Early identification of myocardial microstructural alterations in hypertrophic cardiomyopathy with *in vivo* cardiac diffusion-tensor imaging. Radiol Cardiothorac Imaging. (2025) 7(1):e240009. 10.1148/ryct.240009 39745326

[51] van Ooij P Allen BD Contaldi C Garcia J Collins J Carr J 4D Flow MRI and T1 -mapping: assessment of altered cardiac hemodynamics and extracellular volume fraction in hypertrophic cardiomyopathy. J Magn Reson Imaging. (2016) 43(1):107–14. 10.1002/jmri.24962 26227419 PMC4850842

[52] Ericsson L Hjalmarsson A Akbar MU Ferdian E Bonini M Hardy B Generalized super-resolution 4D flow MRI—using ensemble learning to extend across the cardiovascular system. IEEE J Biomed Health Inform. (2024) 28(12):7239–50. 10.1109/JBHI.2024.3429291 39012742 PMC11735690

[53] Wang L Wang Y Wang J Xiao M Xi XY Chen BX Myocardial activity at (18)F-FAPI PET/CT and risk for sudden cardiac death in hypertrophic cardiomyopathy. Radiology. (2023) 306(2):e221052. 10.1148/radiol.221052 36219116

[54] Nensa F Bamberg F Rischpler C Menezes L Poeppel TD la Fougere C Hybrid cardiac imaging using PET/MRI: a joint position statement by the European Society of Cardiovascular Radiology (ESCR) and the European Association of Nuclear Medicine (EANM). Eur Radiol. (2018) 28(10):4086–101. 10.1007/s00330-017-5008-4 29717368 PMC6132726

[55] Takeishi Y Masuda A Kubo H Tominaga H Oriuchi N Takenoshita S. Cardiac imaging with (18)F-fluorodeoxyglucose PET/MRI in hypertrophic cardiomyopathy. J Nucl Cardiol. (2017) 24(5):1827–8. 10.1007/s12350-016-0686-x 27743298 PMC5629240

[56] Harms HJ Nesterov SV Han C Danad I Leonora R Raijmakers PG Comparison of clinical non-commercial tools for automated quantification of myocardial blood flow using oxygen-15-labelled water PET/CT. Eur Heart J Cardiovasc Imaging. (2014) 15(4):431–41. 10.1093/ehjci/jet177 24107905

[57] Goldie FC Lee MMY Coats CJ Nordin S. Advances in multi-modality imaging in hypertrophic cardiomyopathy. J Clin Med. (2024) 13(3):842. 10.3390/jcm13030842 38337535 PMC10856479

[58] Fahmy AS Rowin EJ Arafati A Al-Otaibi T Maron MS Nezafat R. Radiomics and deep learning for myocardial scar screening in hypertrophic cardiomyopathy. J Cardiovasc Magn Reson. (2022) 24(1):40. 10.1186/s12968-022-00869-x 35761339 PMC9235098

[59] Wang J Bravo L Zhang J Liu W Wan K Sun J Radiomics analysis derived from LGE-MRI predict sudden cardiac death in participants with hypertrophic cardiomyopathy. Front Cardiovasc Med. (2021) 8:766287. 10.3389/fcvm.2021.766287 34957254 PMC8702805

[60] Nakamori S Amyar A Fahmy AS Ngo LH Ishida M Nakamura S Cardiovascular magnetic resonance radiomics to identify components of the extracellular matrix in dilated cardiomyopathy. Circulation. (2024) 150(1):7–18. 10.1161/CIRCULATIONAHA.123.067107 38808522 PMC11216881

[61] Qiu J Li L Wang S Zhang K Chen Y Yang S MyoPS-Net: myocardial pathology segmentation with flexible combination of multi-sequence CMR images. Med Image Anal. (2023) 84:102694. 10.1016/j.media.2022.102694 36495601

[62] Popescu DM Abramson HG Yu R Lai C Shade JK Wu KC Anatomically informed deep learning on contrast-enhanced cardiac magnetic resonance imaging for scar segmentation and clinical feature extraction. Cardiovasc Digit Health J. (2022) 3(1):2–13. 10.1016/j.cvdhj.2021.11.007 35265930 PMC8890075

[63] Captur G Bhandari A Bruhl R Ittermann B Keenan KE Yang Y T(1) mapping performance and measurement repeatability: results from the multi-national T(1) mapping standardization phantom program (T1MES). J Cardiovasc Magn Reson. (2020) 22(1):31. 10.1186/s12968-020-00613-3 32375896 PMC7204222

[64] Greulich S Seitz A Herter D Gunther F Probst S Bekeredjian R Long-term risk of sudden cardiac death in hypertrophic cardiomyopathy: a cardiac magnetic resonance outcome study. Eur Heart J Cardiovasc Imaging. (2021) 22(7):732–41. 10.1093/ehjci/jeaa423 33458753 PMC8219365

[65] Akita K Suwa K Ohno K Weiner SD Tower-Rader A Fifer MA Detection of late gadolinium enhancement in patients with hypertrophic cardiomyopathy using machine learning. Int J Cardiol. (2025) 421:132911. 10.1016/j.ijcard.2024.132911 39706305 PMC11725445

[66] Nauffal V Di Achille P Klarqvist MDR Cunningham JW Hill MC Pirruccello JP Genetics of myocardial interstitial fibrosis in the human heart and association with disease. Nat Genet. (2023) 55(5):777–86. 10.1038/s41588-023-01371-5 37081215 PMC11107861

[67] Zhao K Zhu Y Chen X Yang S Yan W Yang K Machine learning in hypertrophic cardiomyopathy: nonlinear model from clinical and CMR features predicting cardiovascular events. JACC Cardiovasc Imaging. (2024) 17(8):880–93. 10.1016/j.jcmg.2024.04.013 39001729

[68] Masri A Cardoso RN Abraham TP Claggett BL Coats CJ Hegde SM Effect of aficamten on cardiac structure and function in obstructive hypertrophic cardiomyopathy: SEQUOIA-HCM CMR substudy. J Am Coll Cardiol. (2024) 84(19):1806–17. 10.1016/j.jacc.2024.08.015 39217563

[69] Saberi S Cardim N Yamani M Schulz-Menger J Li W Florea V Mavacamten favorably impacts cardiac structure in obstructive hypertrophic cardiomyopathy: EXPLORER-HCM cardiac magnetic resonance substudy analysis. Circulation. (2021) 143(6):606–8. 10.1161/CIRCULATIONAHA.120.052359 33190524

[70] Arbelo E Protonotarios A Gimeno JR Arbustini E Barriales-Villa R Basso C 2023 ESC guidelines for the management of cardiomyopathies. Eur Heart J. (2023) 44(37):3503–626. 10.1093/eurheartj/ehad194 37622657

[71] Wang J Zhang J Pu L Qi W Xu Y Wan K The prognostic value of left ventricular entropy from T1 mapping in patients with hypertrophic cardiomyopathy. JACC Asia. (2024) 4(5):389–99. 10.1016/j.jacasi.2024.01.005 38765656 PMC11099820

[72] Tian Z Li L Li X Wang J Zhang Q Li Z Effect of mavacamten on Chinese patients with symptomatic obstructive hypertrophic cardiomyopathy: the EXPLORER-CN randomized clinical trial. JAMA Cardiol. (2023) 8(10):957–65. 10.1001/jamacardio.2023.3030 37639259 PMC10463173

[73] Tian Z Li L Li X Zhang Q Peng D Ma W Effects of mavacamten on cardiac magnetic resonance features in Chinese patients with obstructive hypertrophic cardiomyopathy. JACC Asia. (2025) 5(8):1064–74. 10.1016/j.jacasi.2025.05.015 40632050 PMC12426844

[74] Khan HR Rodwell P Taha AH Goha A Ahmed M Thain AP Magnetic resonance left ventricle mass-index/fibrosis: long-term predictors for ventricular arrhythmia in hypertrophic cardiomyopathy-a retrospective registry. J Cardiovasc Dev Dis. (2023) 10(3):120. 10.3390/jcdd10030120 36975884 PMC10051998

[75] Song J Chen P Pan X Chen B Zang J Zhang J. Meta-analysis of the association between left-ventricular late gadolinium enhancement on cardiac MRI and atrial fibrillation in patients with hypertrophic cardiomyopathy. Echocardiography. (2025) 42(4):e70144. 10.1111/echo.70144 40152934

[76] Pan J Ng SM Neubauer S Rider OJ. Phenotyping heart failure by cardiac magnetic resonance imaging of cardiac macro- and microscopic structure: state of the art review. Eur Heart J Cardiovasc Imaging. (2023) 24(10):1302–17. 10.1093/ehjci/jead124 37267310 PMC10531211

[77] Bissell MM Raimondi F Ait Ali L Allen BD Barker AJ Bolger A 4D Flow cardiovascular magnetic resonance consensus statement: 2023 update. J Cardiovasc Magn Reson. (2023) 25(1):40. 10.1186/s12968-023-00942-z 37474977 PMC10357639

